# Genetic evidence suggests that GIS functions downstream of TCL1 to regulate trichome formation in *Arabidopsis*

**DOI:** 10.1186/s12870-018-1271-z

**Published:** 2018-04-13

**Authors:** Na Zhang, Li Yang, Sha Luo, Xutong Wang, Wei Wang, Yuxin Cheng, Hainan Tian, Kaijie Zheng, Ling Cai, Shucai Wang

**Affiliations:** 1 Key Laboratory of Molecular Epigenetics of MOE, Northeast Normal University, Changchun, 130024 China; 2 Key Laboratory of Soybean Molecular Design Breeding, Northeast Institute of Geography and Agroecology, Chinese Academy of Sciences, Changchun, China

**Keywords:** GIS, TCL1, Trichome formation, Transcription factor, Arabidopsis

## Abstract

**Background:**

Trichome formation in Arabidopsis is regulated by a MBW complex formed by MYB, bHLH and WD40 transcriptional factors, which can activate *GLABRA2* (*GL2*) and the R3 MYB transcription factor genes. GL2 promotes trichome formation, whereas R3 MYBs are able to block the formation of the MBW complex. It has been reported that the C2H2 transcription factor GIS (GLABROUS INFLORESCENCE STEMS) functions upstream of the MBW activator complex to regulate trichome formation, and that the expression of TCL1 is not regulated by the MBW complex. However, *gis* and the R3 MYB gene mutant *tcl1* (*trichomeless 1*) have opposite inflorescence trichome phenotypes, but their relationship in regulating trichome formation remained unknown.

**Results:**

By generating and characterization of the *gis tcl1* double mutant, we found that trichome formation in the *gis tcl1*double and the *tcl1* single mutants were largely indistinguishable, but the trichome formation in the *35S:TCL1*/*gis* transgenic plant was similar to that in the *gis* mutant. By using quantitative RT-PCR analysis, we showed that expression level of *GIS* was increased in the triple mutant *tcl1 try cpc*, but the expression level of *TCL1* was not affected in the *gis* mutant. On the other hand, trichome morphology in both *gis tcl1* and *35S:TCL1*/*gis* plants was similar to that in the *gis* mutant.

**Conclusions:**

In summary, our results indicate that GIS may work downstream of TCL1 to regulate trichome formation, and GIS has a dominant role in controlling trichome morphology.

## Background

Trichome formation in Arabidopsis has been shown to be a good model for the investigation of plant cell fate determination [16, 20, 21, 23, 34]. Accumulated evidence suggest that trichome formation in Arabidopsis is mainly regulated by several key transcription factors, including the WD40-repeat protein TTG1 (TRANSPARENT TESTA GLABRA1) [31], the R2R3 MYB transcription factor GL1 (GLABRA1) [14], the bHLH transcription factors GL3 (GLABRA3) and EGL3 (ENHANCER OF GLABRA3) [15, 42], the homeodomain protein GL2 (GLABRA2) [18], and several R3 MYB transcription factors [34, 35]. Genetic and molecular evidence indicates that TTG1, GL1, GL3 or EGL3 and GL2 positively regulate trichome formation [14, 15, 18, 31, 42], whereas the R3 MYB transcription factors including TRY (TRIPTYCHON), CPC (CAPRICE), TCL1 (TRICHOMELESS 1), TCL2, ETC1 (ENHANCER OF TRY AND CPC 1), ETC2 and ETC3 negatively regulate trichome formation [5, 10, 11, 19, 22, 28–30, 35, 36, 39].

It has been proposed that TTG1 and GL1 interacted with GL3 or EGL3 to form a MBW (MYB-bHLH-WD40) transcriptional activator complex,which is able to activate *GL2*, whereas GL2 is required for trichome formation [13, 16, 17, 21]. The same MBW activator complex can also activate the R3 MYB genes *TRY*, *CPC*, *ETC1* and *ETC3*, but not *TCL1*, *TCL2* and *ETC2* [5, 34, 35]. R3 MYBs, in turn, can move from the trichome precursor cells to their surrounding cells, where they compete with GL1 for binding of GL3 or EGL3, resulting in the inhibition of the formation of MBW complex. As a result, *GL2* can not be activated, and trichome formation will be inhibited [4, 8, 9, 16, 19, 21, 34].

In addition to the key transcription factors mentioned above, transcription factors from other families have also been shown to involve in the regulation of trichome formation in Arabidopsis. These transcription factors including the C2H2 proteins GIS (GLABROUS INFLORESCENCE STEMS), GIS2 and GIS3 [6, 7, 24], the ZINC FINGER proteins ZFP5, ZFP6 and ZFP8 [1, 7, 43, 44], the squamosa promoter binding type protein *SPL9* (*SQUAMOSA PROMOTER BINDING PROTEIN LIKE 9*) [40], and the membrane binding NAC protein NTL8 (NTM1(NAC with transmembrane motif1)-like 8) [25]. However, the functions of these transcription factors in regulating trichome formation are achieved by regulating, directly or indirectly the expression of the key transcription factor genes. For instance, GIS, GIS3, ZFP5 and ZFP8 were found to be able to regulate some MBW genes [6, 7, 24, 43, 44]. On the other hand, SPL9 and NTL8 have been reported to be able to activate R3 MYB genes *TRY* and *TCL1* [25, 40], although SPL9 and NTL8 may function in different pathway to regulate the expression of *TRY* and *TCL1* [25].

Accumulated evidence in recent years indicates that trichome formation regulating transcription factor regulatory network is much more complicated than previously thought. For example, it had been reported that the expression of *GL1* was directly suppressed by the R3 MYB transcription factor TCL1 [36]. The conserved motif in the R3 domain of GL1 that is required for its interaction with GL3, has recently been shown to involve in the binding of the GL1 to its target genes [2]. It has also been reported that R3 MYB proteins may regulate trichome formation at the absence of GL2 [32].

Previous genetic evidence suggests that GIS may function upstream of the MBW complex in regulating trichome formation in Arabidopsis [6]. However, *gis* and *tcl1* mutants showed a opposite inflorescence trichome phenotypes [6, 36], suggesting that GIS and TCL1 may function in the same pathway in regulating trichome formation. By using genetic and molecular techniques, we dissected the relationship between GIS and TCL1, we found that GIS may function downstream of TCL1 to regulate trichome formation in Arabidopsis.

## Results

### Ectopic trichome formation in the *gis tcl1* double mutant is similar to that in the *tcl1* mutant

Previously we showed that in the *tcl1* mutant, ectopic trichomes were produced on the upper part of the inflorescence including stem internodes and pedicels, whereas the *35S:TCL1* transgenic plants showed a glabrous phenotype [36]. The *gis* single mutant, on the contrary, produced fewer trichomes on stems of the upper inflorescence, but the *35S:GIS* transgenic plants have more stem trichomes [6]. The opposite phenotypes observed in the *tcl1* and *gis* mutants indicate that GIS and TCL1 may have opposite functions in controlling trichome formation in Arabidopsis.

Previously researches showed that GIS was able to affect the expression of some MBW complex genes including the R2R3 MYB gene *GL1*, and the bHLH genes *GL3* and *EGL3* [6]. Considering that some R3 MYB transcription factor genes are regulated by the MBW complex, it is reasonable to assume that GIS may affect the expression of those R3 MYB genes. However, our previously results have shown that the expression *TCL1* was not activated by the MBW complex [35], and TCL1 was able to repress the expression of *GL1*. Thus it will be of great interesting to examine whether GIS and TCL1 may coordinate to regulate trichome formation in Arabidopsis.

**Fig. 1:**
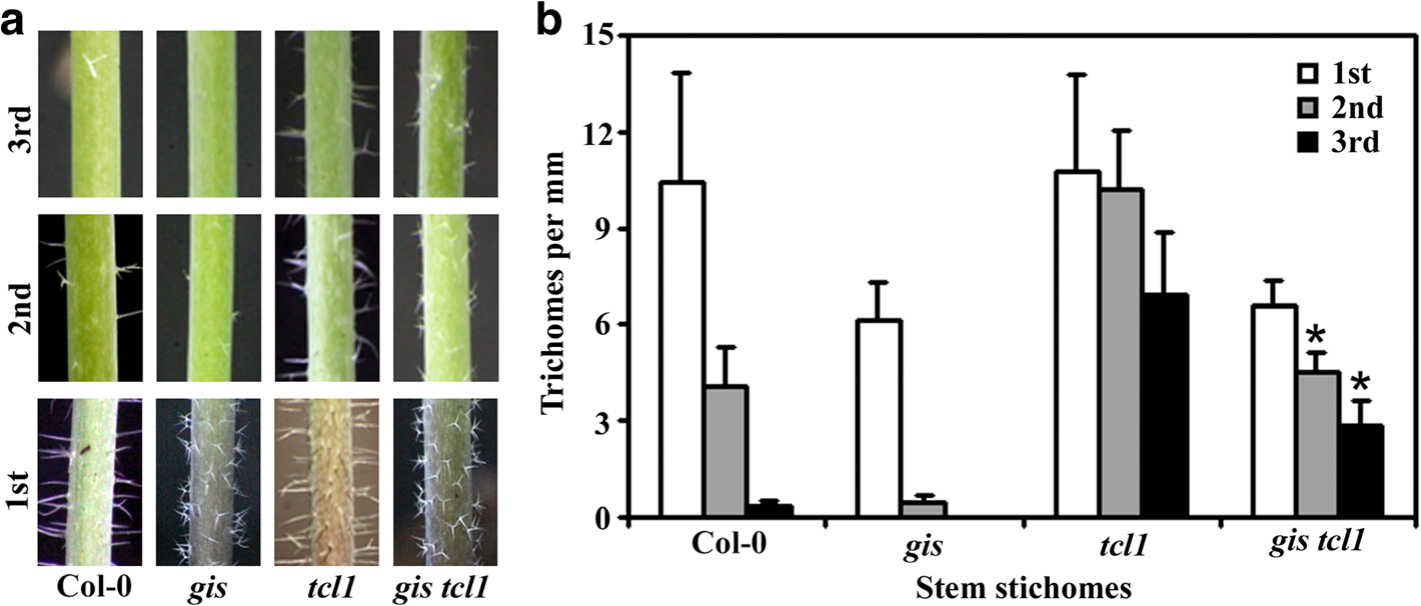
Trichome formation on stems of Col-0 wild type and the *gis*, *tcl1* and *gis tcl1* mutant plants. (**a**) Trichomes on the main stem internodes of the Col-0 wild type and the *gis*, *tcl1* and *gis tcl1* mutants. Photographs were taken from the first three internodes on the main inflorescence stems of 5-week-old soil-grown plants. Note that the trichome patterning on the main inflorescence stem of the *gis tcl1* mutant was similar to that of the *tcl1* mutant, but the morphology of the trichomes on the main inflorescence stem of the *gis tcl1* mutant was similar to that of the *gis* mutant. (**b**) Trichome density on the first three internodes on the main inflorescence stems of the Col-0 wild type and the *gis*, *tcl1* and *gis tcl1* mutants. Number of trichomes on the each internode was count, the length of the internodes was measured, and the trichome density was calculated*.* Data represent the mean ± standard deviation (SD) of 10 plants. *Significantly different form that in the *gis* mutant plants (*P* < 0.0001)

**Fig. 2:**
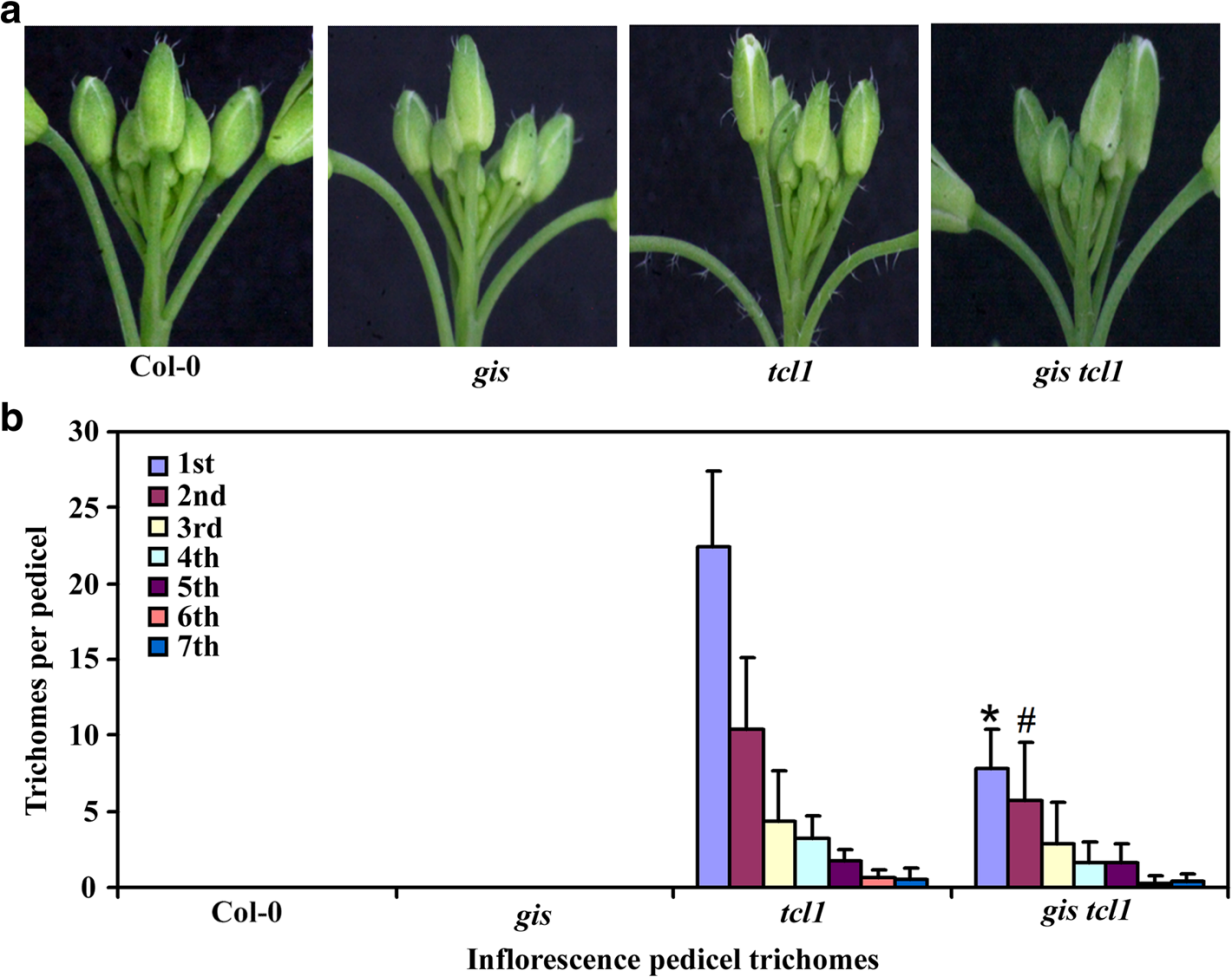
Trichome formation on inflorescences of the Col-0 wild type and the *gis*, *tcl1* and *gis tcl1* mutant plants. (**a**) Trichomes on the main inflorescences of the Col-0 wild type and the *gis*, *tcl1* and *gis tcl1* mutants. Photographs were taken from the main inflorescences of 5-week-old soil-grown plants. (**b**) Trichome numbers on the main inflorescence pedicel of the Col-0 wild type and the *gis*, *tcl1* and *gis tcl1* mutants. Trichomes on the first seven pedicels of the main inflorescence were count. Data represent the mean ± SD of 10 plants. Significantly different form that in the *tcl1* mutant plants (*P < 0.0001, #*P* < 0.05)

To do that, we generated *gis tcl1* double mutant by crossing *gis* and *tcl1* single mutants, and compared trichome phenotypes in the double and the single mutants by growing them side by side. We found that, stem trichome formation in the double mutant *gis tcl1* was largely indistinguishable to that in the single mutant *tcl1* (Fig. 1a). Quantitative analysis showed that numbers of trichomes on the second and third stem internode were greatly decreased in the single mutant *gis* (Fig. 1b), a result similar to reported previously [6], but increased significantly in the single mutant *tcl1* (Fig. 1b). Although all the stem internodes in the double mutant *gis tcl1* produced fewer trichomes when compared with that in the single mutant *tcl1*, the second and third stem internodes in the double mutant *gis tcl1* produced much more trichomes when compared with that in the single mutant *gis* (Fig. 1b). In another word, similar to that in the Col-0 wild type plants, trichomes numbers along the stems decreased sharply in the single mutant *gis*, but slowly in the single mutant *tcl1* and the double mutant *gis tcl1* (Fig. 1). Ectopic trichomes were also found in the pedicels of the *gis tcl1* double mutant (Fig. 2a), although with less numbers when compared with that in the single mutant *tcl1* (Fig. 2b).

**Fig. 3:**
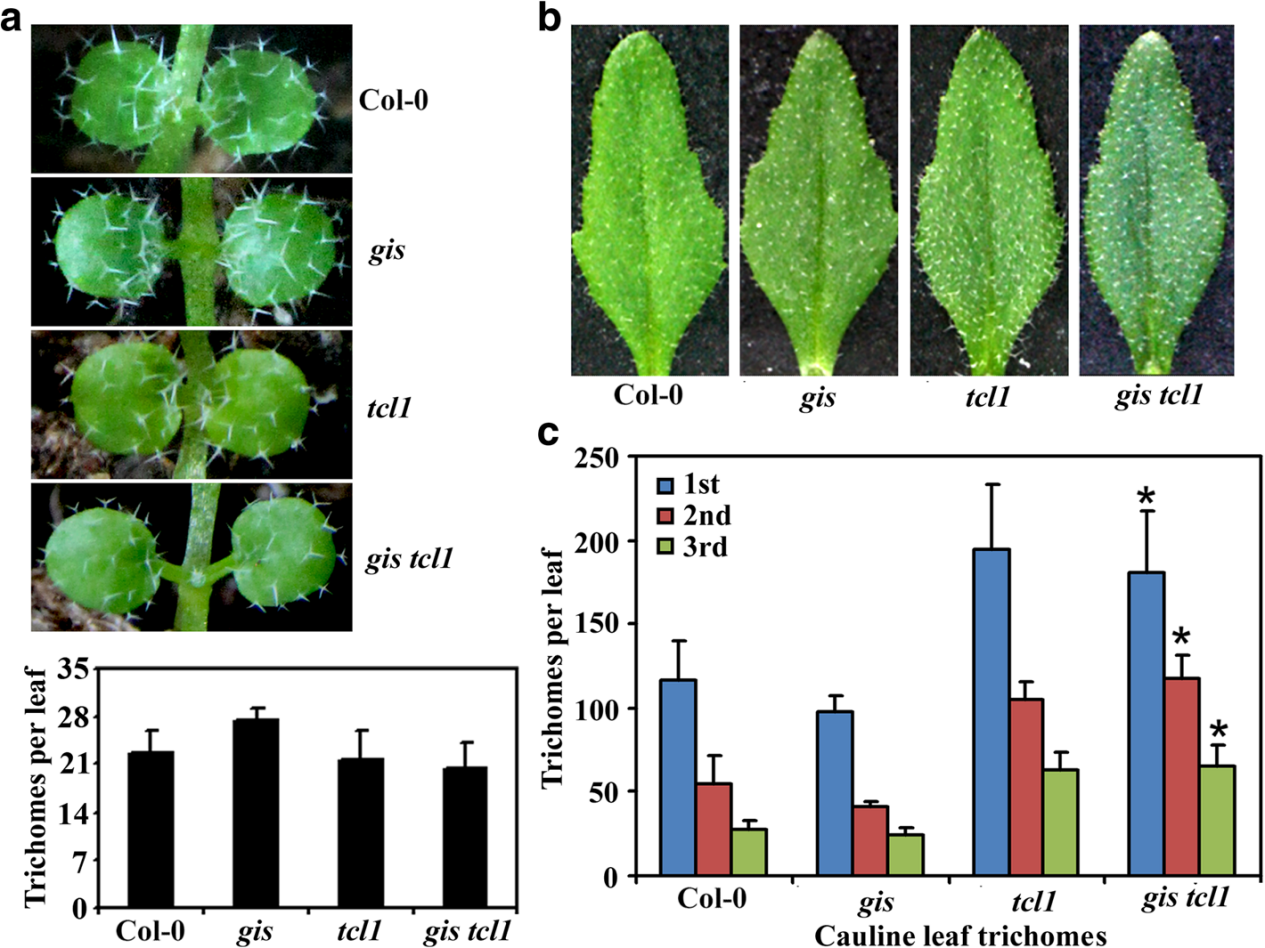
Trichome formation on leaves of Col-0 wild type and the *gis*, *tcl1* and *gis tcl1* mutants. (**a**) Trichomes on the first two rosette leaves of the Col-0 wild type and the *gis*, *tcl1* and *gis tcl1* mutants. Photographs were taken from the first two rosette leaves of 10-day-old soil-grown plants. Graph at the bottom showing the quantification of the trichomes on the first two rosette leaves. Data represent the mean ± SD of 10 plants. (**b**) Trichomes on the first cauline leaves of the Col-0 wild type and the *gis*, *tcl1* and *gis tcl1* mutants. Photographs were taken from the first cauline leaves of 5-week-old soil-grown plants. (**c**) Trichome numbers on the cauline leaves of the Col-0 wild type and the *gis*, *tcl1* and *gis tcl1* mutants. Number of trichomes on the first three cauline leaves was count. Data represent the mean ± SD of 10 plants. *Significantly different form that in the *gis* mutant plants (*P* < 0.0001)

On the other hand, trichome formation on rosette leaves in the single mutants *gis* and *tcl1*, and the double mutant *gis tcl1* was largely similar to that in the Col-0 wild type (Fig. 3a). However, more trichomes were observed on the cauline leaves of the single mutant *tcl1* and double mutant *gis tcl1* when compare with that in the Col-0 wild type and the single mutant *gis* (Fig. 3b, c). Quantitative analysis showed that numbers of trichomes on cauline leaves reduced gradually along the stems in all the plants examined. However, the numbers of trichomes in the single mutant *gis* and Col-0 wild type was similar, whereas that in the double mutant *gis tcl1* and the single mutant *tcl1* was similar (Fig. 3c).

### GIS may function downstream of TCL1 to regulate trichome formation

**Fig. 4:**
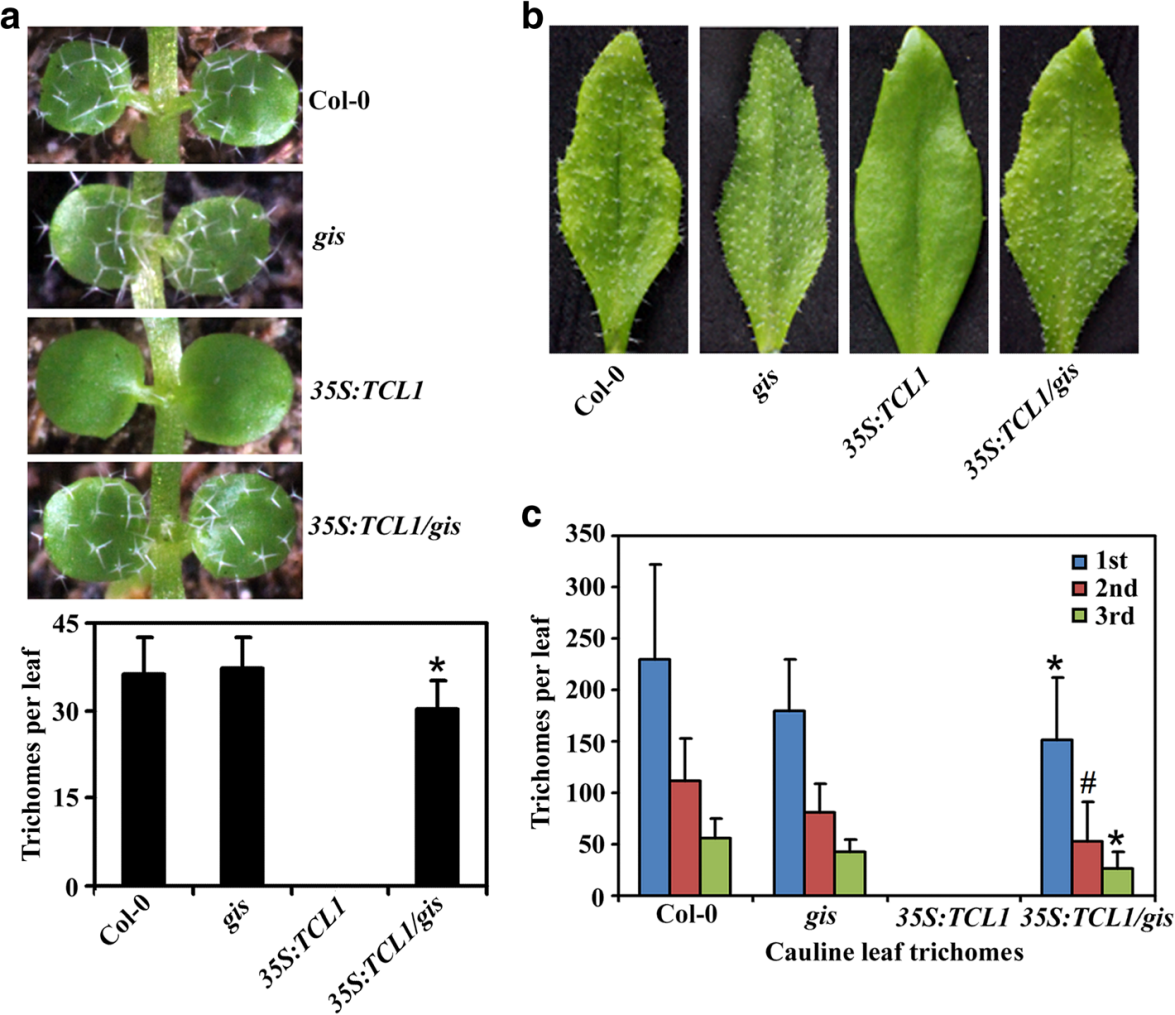
Trichome formation on leaves of Col-0 wild type, the *gis* mutant and the *35S:TCL1* and *35S:TCL1*/*gis* transgenic plants. (**a**) Trichomes on the first two rosette leaves of the Col-0 wild type, the *gis* mutant, and the *35S:TCL1* and *35S:TCL1/gis* transgenic plants. Photographs were taken from the first two rosette leaves of 10-day-old soil-grown plants. Graph at the bottom showing the quantification of the trichomes on the first two rosette leaves. Data represent the mean ± SD of 10 plants. *significantly different form that in the *35S:TCL1* transgenic plants (*P* < 0.0001). (**b**) Trichomes on the first cauline leaves of the Col-0 wild type, the *gis* mutant and the *35S:TCL1* and *35S:TCL1/gis* transgenic plants. Photographs were taken from the first cauline leaves of 5-week-old soil-grown plants. (**c**) Trichome numbers on the cauline leaves of the Col-0 wild type, the *gis* mutant and the *35S:TCL1* and *35S:TCL1/gis* transgenic plants. Number of trichomes on the first three cauline leaves was count. Data represent the mean ± SD of 10 plants. Significantly different form that in the *35S:TCL1* transgenic plants (*P < 0.0001, #*P* < 0.0005)

The above results indicate that GIS and TCL1 may coordinate to regulate trichome formation in Arabidopsis. However, since GIS positively, whereas TCL1 negatively regulate trichome formation [6, 36], it may seems difficult to judge from the above results the functional sequence of GIS and TCL1 in regulating trichome formation. We thus generated *35S:TCL1* transgenic plant in the *gis* mutant background (*35S:TCL1*/*gis*) by crossing the *35S:TCL1* transgenic with the *gis* single mutant plants, and compared trichome formation in the transgenic plant with the *35S:TCL1* transgenic plant and the *gis* single mutant by growing them side by side. As shown in Fig. 4a, the *35S:TCL1* transgenic seedlings showed a glabrous phenotype, a result similar to reported previously [36]. However, trichome production was resumed in the *35S:TCL1*/*gis* transgenic seedlings, to a level similar to that in the Col-0 wild type or the *gis* single mutant seedlings (Fig. 4a).

Observation of mature plants showed that cauline leaves of the *35S:TCL1* transgenic plant failed to produce any trichomes, however, that of the *35S:TCL1*/*gis* transgenic plant were able to do so, similar to that of the Col-0 wild type or the single mutant *gis* (Fig. 4b). Quantitative analysis showed that trichome numbers on cauline leaves of the *35S:TCL1*/*gis* transgenic plants and the Col-0 wild type or the single mutant *gis* were largely similar (Fig. 4c).

**Fig. 5:**
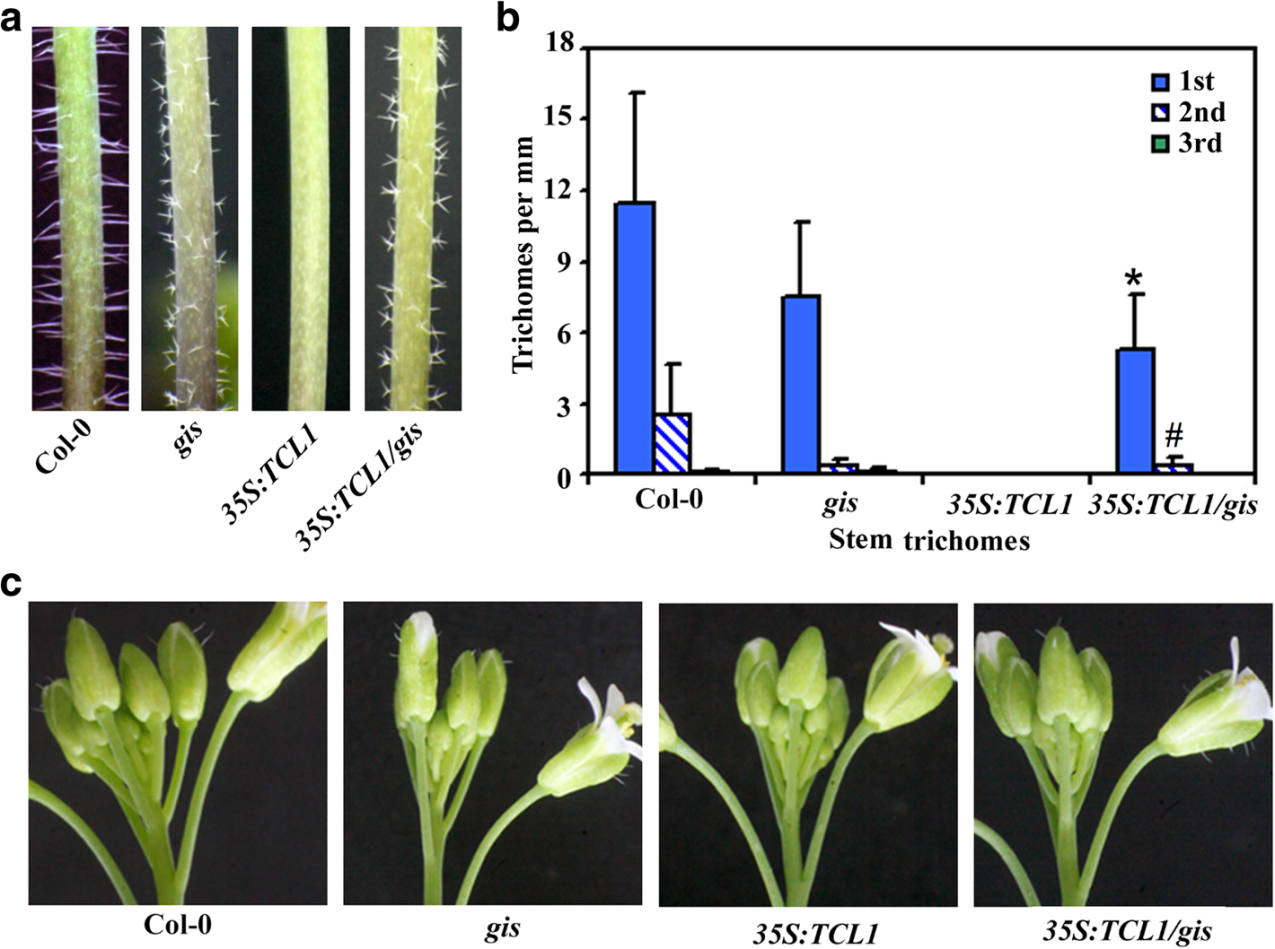
Trichome formation on main inflorescences of Col-0 wild type, the *gis* mutant and the *35S:TCL1* and *35S:TCL1/gis* transgenic plants. (**a**) Trichomes on the main inflorescence stems of the Col-0 wild type, the *gis* mutant and the *35S:TCL1* and *35S:TCL1/gis* transgenic plants. Photographs were taken from the main inflorescence stems of 5-week-old soil-grown plants. Note that the trichome patterning and the morphology of the trichomes on the main inflorescence stem of the *35S:TCL1/gis* plant was similar to that of the *gis* mutant. (**b**) Trichome density on the internodes of main inflorescence stems in the Col-0 wild type, the *gis* mutant and the *35S:TCL1* and *35S:TCL1/gis* transgenic plants. Number of trichomes on the each internode was count, the length of the internodes was measured, and the trichome density was calculated*.* Data represent the mean ± SD of 10 plants. Significantly different form that in the *35S:TCL1* transgenic plants (*P < 0.0001, #*P* < 0.005) (**c**) Trichomes on the main inflorescences of the Col-0 wild type, the *gis* mutant and the *35S:TCL1* and *35S:TCL1/gis* transgenic plants. Photographs were taken from the main inflorescences of 5-week-old soil-grown plants

Because trichome formation on rosette and cauline leaves in the single mutant *gis* was largely indistinguishable to that in the Col-0 wild type, whereas the *gis* single mutant produced fewer trichomes on the stem internodes of the upper part inflorescence [6], we further examined trichome formation in the inflorescences of the *35S:TCL1*/*gis* transgenic plants. We found that trichome formation in the inflorescences of the *35S:TCL1*/*gis* transgenic plants was also largely indistinguishable from that in the single mutant *gis* (Fig. 5). These results indicate that GIS may function downstream of TCL1 to regulate Arabidopsis trichome formation.

### GIS plays a dominate role in controlling trichome morphology

In addition to regulate trichome formation, GIS also regulates trichome morphology [6]. The single mutant *gis* produced smaller but more branched stem trichomes (Fig. 1a). In contrast, the stem trichomes in the single mutant *tcl1* were morphological similar to that in the Col-0 wild type. Similar to that in the single mutant *tcl1*, the double mutant *gis tcl1* produced more stem trichomes on the internodes of the upper part inflorescence. However, the morphology of the stem trichomes in the double mutant *gis tcl1* was still similar to that in the single mutant *gis* (Fig. 1a).

Similar, even though loss-of-function of *GIS* abolished the inhibitory effectors of *TCL1* on the stem trichome formation in the *35S:TCL1* transgenic plants (Fig. 5a), the stem trichomes of the *35S:TCL1/gis* transgenic plants were morphological similar to that in the *gis* single mutant (Fig. 5a). These observation suggests that GIS plays a dominate role in controlling trichome morphology in Arabidopsis.

### Expression of *GIS* is affected by R3 MYB transcription factors

**Fig. 6:**
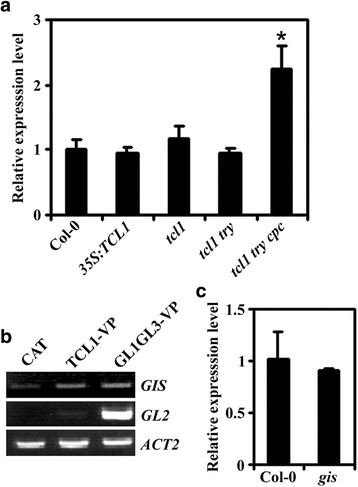
Expression of *GIS* and *TCL1*. (**a**) Expression of *GIS* in seedlings of the Col-0 wild type, the *35S:TCL1* transgenic plant, and the *tcl1*, *tcl1 try* and *tcl1 try cpc* mutants. Total RNA was isolated from 10-day-old seedlings and qRT-PCR was used to examine the expression of *GIS*. *ACT2* was used as an inner control, and the expression level *GIS* in the seedlings of the Col-0 wild type was set as 1. Data represent the mean ± SD of three replicates. *significantly different form that in seedlings of the Col-0 wild type, the *35S:TCL1* transgenic plant, and the *tcl1* and *tcl1 try* mutants (*P* < 0.01). (**b**) Expression of *GIS* in protoplasts transfected with *TCL1-VP* and *GL1GL3-VP*. The plasmids of effector *TCL1-VP* or *GL1GL3-VP* were transfected into protoplasts isolated from rosette leaves of 3- to 4-week-old Col-0 wild type Arabidopsis plants. Contransfection of *CAT* was used a control. The transfected protoplasts were incubated at room temperature in darkness for 20–22 h, then total RNA was isolated and RT-PCR was used to examine the expression of *GIS* and *GL2*. The expression of *ACT2* was used as a control. (**c**) Expression of *TCL1* in the seedlings of the Col-0 wild type and the *gis* mutant. Total RNA was isolated from 10-day-old seedlings and qRT-PCR was used to examine the expression of *TCL1*. *ACT2* was used as an inner control, and the expression level of *TCL1* in seedlings of the Col-0 wild type was set as 1. Data represent the mean ± SD of three replicates

Having shown that GIS may function downstream of TCL1 to regulate Arabidopsis trichome formation, we wanted to examine whether TCL1 may affect the expression of *GIS*. Total RNA isolated from seedlings of the *35S:TCL1* transgenic plant and the single mutant *tcl1* was used to check the transcript level of *GIS* by quantitative RT-PCR. As shown in Fig. 6a, the expression level of *GIS* in both the *35S:TCL1* transgenic and the single mutant *tcl1* seedlings was similar to that in the Col-0 wild type seedlings. It has been reported that R3 MYBs function redundantly to regulate Arabidopsis trichome formation [35], we thus further checked whether the expression of *GIS* in the seedlings of the double mutant *tcl1 try* and the triple mutant *tcl1 try cpc* may be affected. We found that although no changes in the expression level of *GIS* was observed in the double mutant *tcl1 try*, an ~ 2 folds increase was observed in the triple mutant *tcl1 try cpc* (Fig. 6a), indicating that R3 MYBs may have redundant function in regulating the expression of *GIS*.

Considering that GIS functions down stream of TCL1 to regulate trichome formation, and the expression level of GIS was increased in the tcl1 try cpc triple mutant, we wanted to examine whether TCL1 may directly regulate the expression of *GIS*. To do that, we decided to use protoplast transient transfection assays. Previously we have successfully used these assays combined with RT-PCR or qRT-PCR analysis to examine the activation of endogenous genes by several different transcription factors [33, 35, 37], and we have shown that TCL1 functioned as a transcription activator when fused with VP16 (TCL1-VP), a strong transcription activator [36]. We thus transfected plasmid DNA of the *TCL1-VP* construct into Arabidopsis protoplasts and examined the expression of *GIS* by RT-PCR. As shown in Fig. 6b, transfection of *TCL1-VP* only slightly increased the expression of *GIS*. As a control, transfection of *GL1GL3-VP* greatly activated the expression of *GL2*, a result has been reported previously [33].

We also examined whether the expression of *TCL1* may be affected by GIS, by examining the transcript level of *TCL1* in the single mutant *gis*. We found that the transcript level of *TCL1* in the single mutant *gis* remained largely unchanged when compared that in the Col-0 wild type (Fig. 6c).

## Discussion

The seven Arabidopsis R3 MYBs showed highly similarity at amino acid level [34], they all have [D/E]Lx2[R/K]x3Lx6Lx3R, the amino acid signature that is required for protein-protein interaction of MYB with bHLH proteins [41], and WxM, a motif has been shown to be required for cell-to-cell movement of the R3 MYB protein CPC [12]. Consistent with these features, all the seven R3 MYB transcription factors were able to interact with GL3/EGL3 in plant cells [5, 35], over-expression any of the R3 MYB genes resulted in glabrous phenotypes in Arabidopsis [5, 19, 22, 29, 30, 36], and characterization of the double, triple and higher order mutants of the R3 MYB genes indicated that R3 MYBs functioned in a highly redundant manner in regulating trichome formation in Arabidopsis ([3–5, 10, 11]; Tominage et al. 2008; [35, 36, 39]).

Among the seven R3 MYBs, TCL1 is unique in several different aspects. First, in addition to interact with GL3/EGL3, TCL1 has been shown to be able to directly suppress the R2R3 MYB gene *GL1* [36]. Second, unlike *TRY*, *CPC*, *ETC1* and *ETC3*, the expression of *TCL1* was not regulated by the MBW activator complex [5]. Third, TCL1 has been shown to be a direct target gene of SPL9 and NTL8 [25, 40]. We provide evidence in this study that GIS function downstream of TCL1 in regulating Arabidopsis trichome formation.

According to previously publications, the *gis* and *tcl1* mutants showed opposite phenotypes in stem trichome formation [6, 36], indicating that GIS and TCL1 may function in a same pathway in regulating trichome formation. Therefore a genetic approach was used to dissect the relationship between GIS and TCL1. We found that trichome patterning in the double mutant *gis tcl1* was largely indistinguishable to that in the single mutant *tcl1* (Fig. 1, Fig. 2, Fig. 3). Judged only from this observation, it may seems that TCL1 acts downstream of GIS in regulating trichome formation. However, because GIS positively regulates trichome formation in Arabidopsis [6], where as TCL1 negatively regulate trichome formation [36], it may also reasonable to assume that GIS acts down stream of TCL1. The observation that *35S:TCL1*/*gis* transgenic plant showed a trichome phenotype similar to that in the *gis* mutant (Figure 4, Fig. 5), rather than a glabrous phenotype as observed in the *35S:TCL1* transgenic plants [36], favored the conclusion that GIS functions down stream of TCL1 in regulating trichome formation. Consistent with this, the expression of *GIS* was increased in the triple mutant *tcl1 try cpc*, whereas the expression of *TCL1* in the single mutant *gis* was largely unaffected (Fig. 6).

On the other hand, both the *gis tcl1* double mutant and the *35S:TCL1*/*gis* transgenic plant produced small, more branched trichomes, a phenotype similar to that observed in the *gis* mutant (Figu. 1, Fig. 4), suggesting that GIS functions dominantly in controlling trichome morphology.

It should be noted that although the expression of *GIS* was increased in the *tcl1 try cpc* mutant, its expression level in the *tcl1* mutant and the *35S:TCL1* transgenic plants remained largely unchanged (Fig. 6). Together with the observation that tranfection of *TCL1-VP* into Arabidopsis only slightly increased the expression level of *GIS*, our results indicate that it is unlikely that TCL1 can directly regulate the expression of *GIS*.

It has been shown that the expression of *TCL1* is directly regulated by SPL9 and NTL8 [25, 40], whereas TCL1 is able to regulate *GL1* expression directly [36]. Even though our results could not support a role of TCL1 in regulating *GIS* expression, the observation that GIS function downstream of TCL1 added another regulation loop into the transcription factor regulating networks that control trichome formation in Arabidopsis.

## Conclusions

GIS acts downstream of TCL1, and possible other R3 MYB proteins to regulate trichome formation, and GIS play a dominant role in regulating trichome morphology.

## Methods

### Plant materials used in this study and growth conditions

The wild type Arabidopsis ecotype Col-0 was used as a control in phenotypic assays and for protoplast isolation. The single mutants *gis* and *tcl1*, double mutant *tcl1 try*, triple mutant *tcl1 try cpc* and the *35S:TCL1* transgenic plants in the Col-0 background have been reported previously [6, 35, 36]. The double mutant *gis tcl1* was obtained by crossing the single mutants *gis* with *tcl1*, examining the putative mutant phenotypes in the F_2_ progeny, and confirming their double mutant status by genotyping. The *35S:TCL1*/*gis* transgenic plant was obtained by crossing the *35S:TCL1* transgenic plant with the *gis* mutant, examining the putative mutant and transgenic plant phenotypes in the F_2_ progeny, and confirming the *gis* mutant and *35S:TCL1* overexpression status by genotyping.

For RNA isolation, sterilized seeds were grown on 1/2 MS (Murashige & Skoog) plates with vitamins (Plantmedia) and 1% (*w*/*v*) sucrose, and solidified with 0.6% phytoagar (Plantmedia). To obtain plants for phenotypic analysis and protoplasts isolation, Arabidopsis seeds were sown directly into soil filled pots. All the Arabidopsis plants were growth in a growth room with a temperature at 22 °C and a 16/8 h photoperiod with light density at about 120 μmol m^− 2^ s^− 1^.

### RNA isolation, PCR and quantitative RT-PCR (qRT-PCR)

Total RNA from 10-day-old Arabidopsis seedlings was isolated by using EasyPure Plant RNA Kit and following the instruction provided by the manufacturer (TransGen Biotech). Two μg RNA was subjected to complementary DNA (cDNA) synthesis via reverse transcription primed by Oligo(dT). The cDNA was synthesized by using DNA Synthesis Super Mix provided by TransGene Biotech in a reaction volume of 20 μl. For qRT-PCR, the synthesized cDNA was diluted 10 times with TE buffer (pH = 8.0), and 1 μl of the dilute solution was then used for each PCR reaction. The qRT-PCR was carried out on an Applied Biosystems StepOnePlus™ Real-Time PCR system by using the TransStart™ Top Green qPCR SuperMix reagent (TransGen Biotech). Total RNA from transfected protoplasts was isolated, and cDNA was synthesized by following the procedures described previously [37], with exceptions that an EasyPure Plant RNA Kit was used for RNA isolation, and EasyScript First-Strand DNA Synthesis Super Mix was used for cDNA synthesis.

The primers used to examine the expression of *GIS*, *TCL1*, *GL2* and *ACT2* have been reported previously [6, 25, 45].

### Constructs

The reporter construct *Gal4-GUS* and the effect constructs *GD* (*Gal4 DNA binding domain*), *GD-VP*, *GD-SPL9*, *TCL1-VP*, *GL1GL3-VP*, and *CAT* used for protoplasts transfection have been reported previously [25–27, 33, 36].

### Plasmid DNA preparation, Arabidopsis protoplast isolation, protoplast transfection

Plasmid DNA of the effector was isolated using the GoldHi EndoFree Plasmid Maxi Kit by following the procedures provided by the manufacturer (Kangwei).

Protoplasts were isolated and transfected by following the procedures described previously [37, 38]. Briefly, rosette leaves were collected from 3- to 4-week-old Col-0 wild type plants grown in soil pots and used for protoplast isolation. Isolated protoplasts were transfected with plasmid DNA isolated. The protoplasts were then incubated in darkness for 20–22 h at room temperature, and then subjected to RNA isolation, or GUS activity assays on a BioTEK Synergy™ HT microplate reader.

### Microscopy

Trichome formation and trichome morphology in Arabidopsis plants was analyzed under a microscope (Motic K) and photographed with a digital camera (EOS 1100D) attached to the microscope. Trichome formation on rosette leaves was observed using the first two true leaves of the wild type and mutant seedlings grown on soil pots. Trichome formation on inflorescences and cauline leaves was observed using adult soil-grown plants.
